# Comparison of different diagnostic protocols for the detection of *Toxocara* spp. in faecal samples of cats and dogs

**DOI:** 10.1186/s13071-024-06524-x

**Published:** 2024-10-24

**Authors:** Deliah Tamsyn Winterfeld, Birgit Schauer, Majda Globokar, Nikola Pantchev, Susan Mouchantat, Franz Josef Conraths, Helge Kampen, Johanna Dups-Bergmann, Gereon Schares, Pavlo Maksimov

**Affiliations:** 1 Institute of Epidemiology, Friedrich-Loeffler-Institute, Federal Research Institute for Animal Health, Greifswald-Insel Riems, Germany; 2 Institute for Community Medicine, Section SHIP-KEF, University Medicine Greifswald, Greifswald, Germany; 3 IDEXX Laboratories, Kornwestheim, Germany; 4 Institute of Infectology, Friedrich-Loeffler-Institute, Federal Research Institute for Animal Health, Greifswald-Insel Riems, Germany

**Keywords:** *Toxocara canis*, *Toxocara cati*, Sedimentation-flotation technique, Sequential sieving, DNA extraction, qPCR, Sensitivity

## Abstract

**Background:**

*Toxocara canis* and *Toxocara cati* are parasitic nematodes that occur worldwide. As embryonated *Toxocara* spp. eggs in the environment pose a zoonotic risk, especially for children, optimal diagnostic approaches are necessary for effective disease response and management, including surveillance. However, little is known about the performance of different diagnostic protocols for detecting *Toxocara* spp. in the faeces of cats and dogs, hampering movement towards an optimal diagnostic process. This study aimed to compare detection methods, including a newly developed sequential sieving protocol (SF-SSV) and a high-throughput multiplex qPCR-based method to facilitate epidemiological studies.

**Methods:**

Species-specific *Toxocara* spp. egg suspensions and canine and feline faecal samples from the field were used to estimate analytical and diagnostic sensitivity of the protocols. The performance of two automated DNA extraction protocols using enzymatic and mechanical lysis were compared by multiplex qPCR, targeting both *T. canis* and *T. cati*-specific genomic sequences. All samples were examined by microscopy-based techniques, the sedimentation flotation technique (SF) and a newly developed SF-SSV for the detection, enrichment and purification of parasite eggs. The costs and processing times necessary for all protocols were estimated and compared for both single samples and sets of 100 samples.

**Results:**

To detect *Toxocara* spp. eggs, SF-SSV showed the highest analytical sensitivity and a significantly higher diagnostic sensitivity than the DNA detection methods. Mechanical lysis performed better than enzymatic lysis for automated DNA extraction. In automated DNA extraction, 96-well plates performed better than 24-well plates. DNA detection and microscopy-based parasitological methods showed substantial agreement between the results generated by each method. Microscopy-based techniques required the lowest costs and least hands-on time for a single sample. However, when costs and labour were estimated for a set of 100 samples, the DNA detection protocol using 96-well plates for extraction revealed costs similar to SF-SSV and the fastest processing times.

**Conclusions:**

SF-SSV was superior in terms of analytical and diagnostic sensitivity for the detection of *Toxocara* spp. eggs. For larger sets of samples, multiplex qPCR-based DNA detection represents an alternative to microscopy-based methods, based on the possibility of faster sample processing at similar costs to SF-SSV, and the ability to provide species-specific diagnoses.

**Graphical Abstract:**

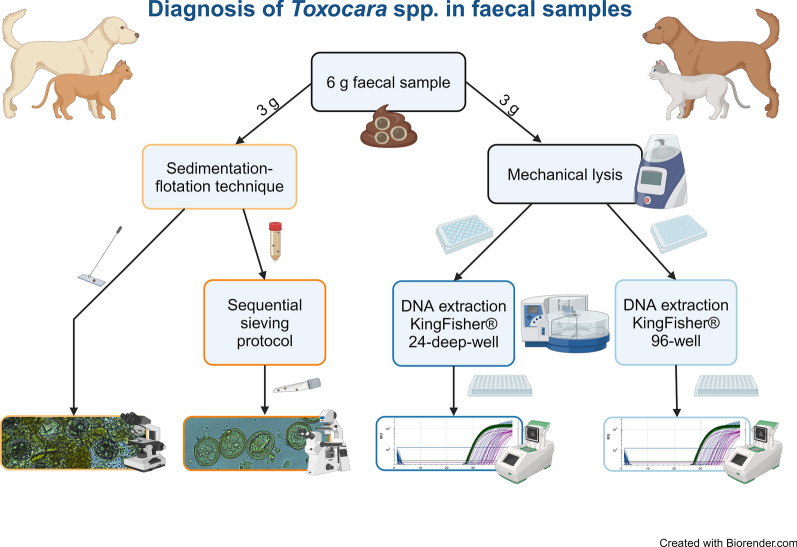

**Supplementary Information:**

The online version contains supplementary material available at 10.1186/s13071-024-06524-x.

## Background

*Toxocara canis* and *T. cati* are worldwide occurring nematodes. Embryonated *Toxocara* spp. eggs in the environment pose a zoonotic risk, especially for children [1]. The course of the disease and its clinical signs or symptoms vary. They can include fever, blindness and other symptoms resulting from damage to organs by migrating larvae [2].

Cats and dogs are definitive hosts of *T. cati* and *T. canis*, respectively. Infection of these animals results in the shedding of helminth eggs into the environment, which varies quantitatively with age, particularly in dogs, with puppies exhibiting a markedly higher faecal egg count than adult animals [1]. Therefore, cats and dogs play a primary role in the epidemiology of these zoonotic parasites [1, 3]. After the eggs have embryonated, they serve as a source of infection not only for definitive and intermediate hosts (different species of small rodents, other mammals, avian species and invertebrates) but also for aberrant hosts (e.g. humans) [3]. Under optimal conditions *Toxocara* spp. eggs are thought to survive for up to 4 years in the environment [1]. Human infections usually occur by oral uptake of infectious embryonated eggs via either consumption of contaminated vegetables [4, 5] or ingestion of contaminated soil while being active outdoors [1, 5]. Humans may also become infected following direct contact with contaminated hair [6] or faeces [7] of infected definitive hosts. Children are especially at risk of exposure and thus infection due to their behaviour [1].

The reported prevalence of *Toxocara* spp. in Germany in the years 2003 to 2012 ranged from 0.0% to 3.9% in cats and 0.9% to 6.1% in dogs based on test results obtained with non-randomly sampled faecal samples submitted to diagnostic laboratories [8]. Furthermore, another study reported a mean prevalence of *Toxocara* spp. of 4.6% in dogs and 4.8% in cats during the years 2004–2006 [9]. In 2015–2017, *Toxocara* spp. was detected in 3.8% and 3.5% of faecal samples from dogs and cats, respectively [9]. Other studies based on data from Germany reported average prevalence of 7.7% for cats [10] and 5.9% for dogs [11].

To detect infections in cats or dogs, the sedimentation-flotation technique (SF) is most commonly employed to examine faecal samples for the presence of *Toxocara* spp. eggs. For a combined SF, using zinc chloride solution and “merthiolate-iodine-formalin-concentration”, a diagnostic sensitivity of 87% was reported [12]. For detection of other nematode eggs, i.e. *Toxascaris leonina*, a diagnostic sensitivity of 84% was found for SF, using the Wisconsin double centrifugation technique and Sheather’s sucrose floating solution [13]. Whilst SF is a well-established technique with an acceptable sensitivity, it is limited in that it is time-consuming (especially when large numbers of samples are to be tested) and requires personnel with microscopy experience. Furthermore, species diagnosis, based on morphological characters (size, egg surface), is difficult [14], although differentiating *Toxocara* spp. eggs in faecal samples of cats and dogs by microscopy is theoretically possible [15]. Morphological differentiation is further complicated when samples are frozen because of morphological changes brought about by the freezing and thawing process.

To overcome these limitations, two approaches were taken in this study. First, the possibility of applying available TaqMan^®^-based real-time quantitative PCR (qPCR) assays [16] to detect and differentiate *Toxocara* spp. and to characterize their diagnostic properties was explored. A time-efficient PCR-based high-throughput protocol with potentially comparable or better diagnostic performance relative to SF was then developed and tested for diagnostic performance. As part of this process, different DNA extraction approaches were also investigated.

Second, a sequential sieving method (SF-SSV) was established to enrich *Toxocara* spp. eggs. This method had been shown to be a significantly more sensitive alternative to SF for taeniid eggs and to have a synergistic effect when used in combination with PCR detection of *Echinococcus multilocularis* [17]. Furthermore, by enriching the eggs through sequential sieving, the eggs are cleaned from copro-inhibitors, which may increase their detectability via PCR [17]. Taken together, there was clear potential in this method as a stand-alone diagnostic approach or as an addition to the genomic detection (PCR) approach.

This study reports the establishment of these diagnostic protocols, comparison of their performance against existing diagnostic methods (SF) and the results of their diagnostic validation. Field samples were used to estimate the prevalence of *Toxocara* spp. through the different diagnostic protocols established in this study. Lastly, a comparison of the methods in terms of time and cost is also presented.

## Methods

### Faecal samples

Sample specifications are shown in Table S1 (Additional file 1). A panel of reference faecal samples (*n* = 120), collected as part of routine diagnostic testing, was provided by IDEXX Laboratories Inc. (Kornwestheim, Germany). The faecal samples originated from 38 cats and 82 dogs. All 38 cat samples were positive for *Toxocara* spp. eggs, as determined by SF. Of the 82 dog samples, 71 were *Toxocara* spp. positive and 11 were negative by SF.

In addition, 180 field faecal samples collected within the One Health module [18] of the third cohort (NEXT) of the population-based project Study of Health in Pomerania (SHIP) [19] were included in this study. These samples were collected from 87 cats and 93 dogs living in Mecklenburg Pomerania. Due to the nature of this project, no prior information on the parasitological status of the samples was available.

For biosafety reasons and the possibility of infectious *E. multilocularis* eggs being present in the samples, all field faecal samples were frozen at −80 °C for a minimum of 7 days prior to processing.

### Sedimentation flotation technique

The SF was performed as previously described [20] with a few modifications. Briefly, 3 g of faeces was weighed in a plastic cup, suspended with approximately 20 ml tap water and sieved through a strainer (8–11 mm mesh) into a conical cylinder, which was filled to 250 ml with tap water. Egg suspensions were also sieved through a strainer and processed the same way as the faecal samples. Samples were sedimented overnight, i.e. approximately 15 h, after which the supernatant was sharply decanted and discarded. For each sample, up to 7 ml of the sediment was transferred to a 50-ml centrifuge tube (Sarstedt, Nümbrecht, Germany), which was filled up to 50 ml with a concentrated sugar solution (500 g sugar dissolved in 400 ml water [1.3 g/cm^3^]). Samples were then centrifuged at approximately 1800 *g* for 10 min at room temperature (RT). Following centrifugation, 5 drops (approximately 10 µl) of floated material from the surface of the sample was transferred to a microscope slide using an inoculating loop and covered with a coverslip (22 mm × 22 mm, Menzel-Gläser, Brunswick, Germany). During microscopic examination (20 × magnification), discovered eggs were counted. Remaining supernatants were stored at 4 °C for no longer than 24 h before processing by SF-SSV.

### Sequential sieving

A new protocol for sequential sieving (SF-SSV) was employed based on previous studies [17, 21]. In this approach, the supernatants (approximately 45 ml) from the SF (described above) were sequentially purified through three different sieves (SSV) with 105-, 40- and 20-µm mesh sizes. To remove large particulate matter, the supernatant was first decanted over the 105-µm nylon sieve and the filtrate collected in a beaker. The filtrate was then drawn through a 40-µm nylon mesh to capture matter in the size range of 40 µm–105 µm (*Toxocara* spp. eggs) and subsequently through a 20-µm nylon mesh to capture matter in the size range of 20 µm–40 µm (e.g. fragmented *Toxocara* spp. eggs). To achieve this, the latter two meshes were inserted into reusable syringe filters (Omnilab, Bremen, Germany), and the filtrate was drawn through under negative pressure generated by a vacuum pump (BVC 21, Vacuubrand, Wertheim, Germany). To release captured *Toxocara* spp. eggs from the 40-µm filter, a disposable 50-ml syringe with a Luer lock connector (Braun, Melsungen, Germany) was attached to the filter and 50 ml 0.2% Tween20 solution flushed through into a 50-ml tube (Falcon, Sarstedt, Nümbrecht, Germany). The collected egg suspensions were then centrifuged (1000 *g*, 15 min, RT, without brake), and the resulting supernatant was removed by suction until approximately 5 ml volume including the pellet remained in the tube. The pellet was resuspended in the remaining supernatant, transferred to a 7-ml cell culture tube (Thermofisher Scientific, Waltham, MA, USA) and inspected for the presence of *Toxocara* spp. eggs under the microscope (10 × magnification, Stemi 2000-C, Zeiss, Oberkochen, Germany). Eggs were counted in ten fields per view and an average egg number per field of view was calculated. After microscopy, each sample was centrifuged (1000 *g*, 5 min, RT, without brake), the supernatant removed and the pellet dissolved in 2.5 ml penicillin-streptomycin solution (0.9% sodium chloride, 2% penicillin-streptomycin). All samples were stored at 4 °C.

### DNA isolation

**Fig. 1:**
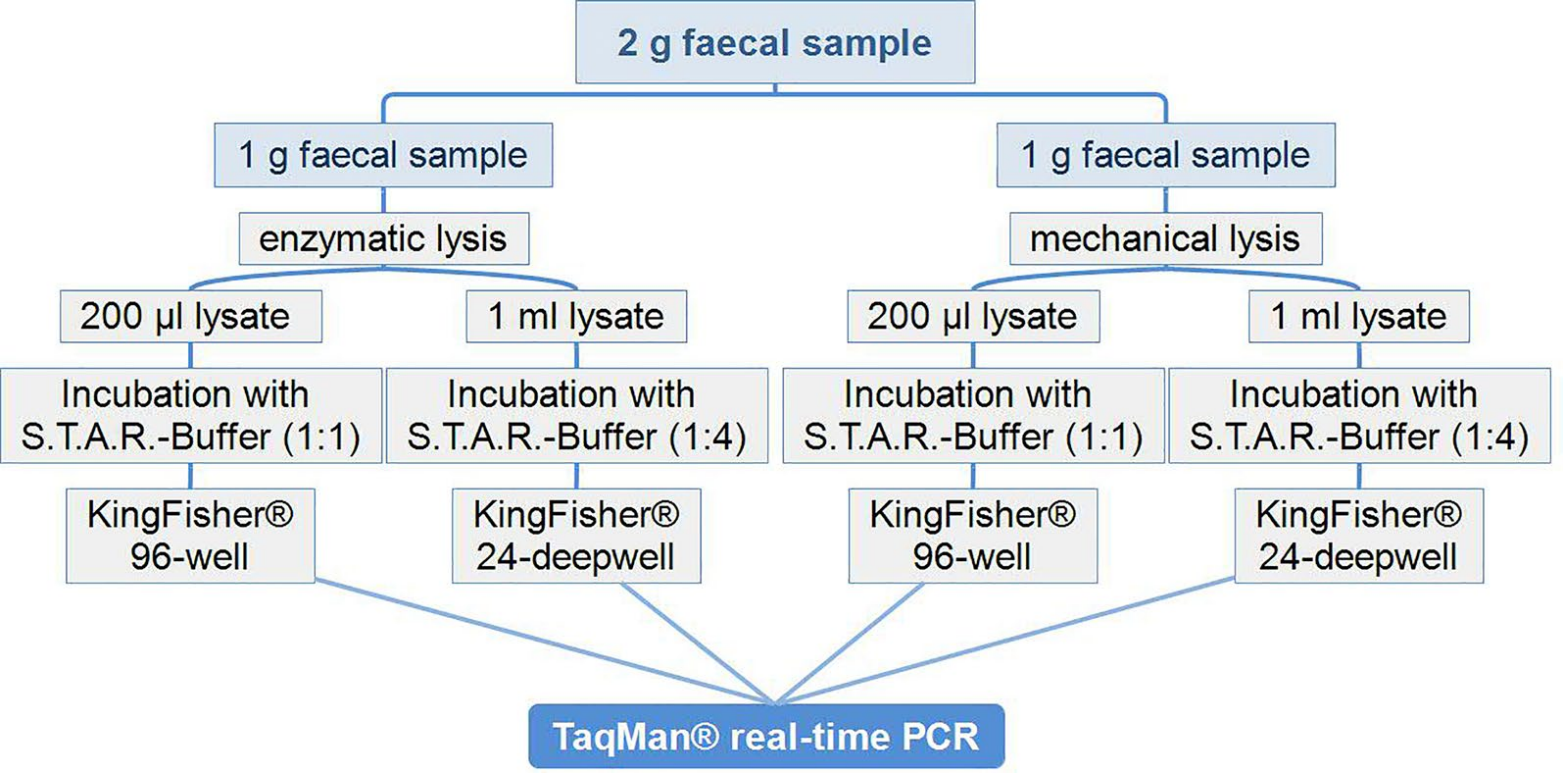
Flowchart of the four extraction protocols applied to extract *Toxocara* spp. DNA from faecal samples

Four automated DNA extraction protocols were used and validated in this study. In the first two protocols, enzymatic lysis, including proteinase K digestion, was applied. In the remaining two extraction procedures, the samples were processed mechanically using the "bead beating" principle (Fig. 1).

Within both the enzymatic and mechanical extraction protocols, two different lysate volumes were used for DNA extraction, i.e. 200 µl or 1 ml lysate (Fig. 1).

For the extraction protocol with enzymatic lysis [22], 1 g of a faecal sample was added to a 15-ml tube with 12 ml lysis buffer (100 mM Tris HCl, pH 8.0, 5 mM EDTA, 0.2% SDS, 200 mM NaCl) and incubated (10 min, 100 °C). After the samples had cooled down to ambient temperature, they were centrifuged (200 *g*, 10 s, RT), and 50 µl proteinase K (20 mg/ml, Qiagen, Hilden, Germany) was added. Samples were then incubated in a water bath with a shaking tray (100 rpm, 2 h, 56 °C; VLSB18, VWR, Darmstadt, Germany), followed by a further incubation step (10 min, 100 °C) to inactivate proteinase K. After the samples had cooled down to ambient temperature, they were centrifuged (15 min, 3500 *g*, RT, without brake) and approximately 10 ml of the resulting supernatant was transferred to a clean 15-ml tube.

For the mechanical lysis protocols, 1 g (for comparison with enzymatic lysis) or up to 3 g (for comparison with SF and SF-SSV) of a faecal sample or egg suspensions was mixed with 0.8 g zirconium oxide beads (diameter 0.5 mm), 0.6 g zirconia beads (diameter 2 mm), 6 ml lysis buffer and 2 ml 5 M sodium chloride solution in a 15-ml tube (Falcon Screw Cap, Sarstedt, Nümbrecht, Germany). The cap was closed tightly and the tube sealed with Parafilm^®^M. The suspension was shredded by FastPrep^®^−24 (MP Bio, Santa Ana CA, USA) four times (60 s, 30.0 Hz.) with 2-min breaks in between. The shredded homogenised samples were subsequently centrifuged (3000 *g*, 10 min, RT, without brake) and the supernatants transferred to clean tubes.

The supernatants from the two lysis protocols described above were then used for automated DNA extraction with a King Fisher^®^ Flex System (Thermo Scientific, Waltham, MA, USA). Two different extraction protocols were tested, a 96-well plate protocol (KF96) and a 24-deep-well plate protocol (KF24).

For KF96, 200 µl of lysate was mixed with 200 µl S.T.A.R. buffer (Roche, Basel, Switzerland) in a 1.5-ml reaction tube (Eppendorf, Hamburg, Germany) and incubated with shaking (500 rpm, 15 min, 95 °C; Thermomixer comfort, Eppendorf, Hamburg, Germany). Following incubation, the samples were vortexed and centrifuged (approximately 24000 *g*, 5 min, 4 °C) and 200 µl of the supernatant processed for automated DNA extraction using the NucleoMag^®^ VET-Kit (Macherey–Nagel, Düren, Germany) as per the supplier’s instructions without using carrier RNA.

For KF24, 1 ml of supernatant was mixed with 3 ml S.T.A.R. buffer in a 5-ml reaction tube (Eppendorf, Hamburg, Germany) or in a 15-ml tube, and the solution was incubated and centrifuged as described for KF96. As for KF96, the NucleoMag^®^ VET-Kit programme and reagents were used, but with the following modifications in volumes: For lysis and binding of 1 ml of the prepared faecal sample, 900 µl VL1 Lysis Buffer, 30 µl proteinase K (75 mg/ml), 50 µl B-Beads and 3 ml VEB Binding Buffer were added. Wash plates were filled with 2 ml wash buffer per well, and the final elution plate was filled with 500 µl VEB elution buffer per well.

### Multiplex TC-qPCR

The multiplex TC-qPCR for the simultaneous detection of *T. canis* and *T. cati* (including an internal control to monitor inhibition of the qPCR) was performed as previously described [23] using QuantiTect multiplex PCR NoROX Master Mix (Qiagen, Hilden, Germany) and undiluted DNA.

### Validation of the protocols

#### Comparison of mechanical and enzymatic lysis

In the first step, the performance of mechanical and enzymatic lysis was compared (Fig. 1). A set of 23 *Toxocara* spp.-positive faecal samples (two cat and 21 dog samples out of the pool of 120 reference faecal samples) from naturally infected animals were used for this purpose (Additional file 1: Table S1). The positivity of the respective samples (from cats and dogs) was confirmed by SF.

#### Analytical sensitivity using egg suspensions

For the analytical sensitivity test, egg suspensions of the respective *Toxocara* spp. were taken from the SF-SSV of highly positive samples (> 100 eggs in SF; Additional file 1: Table S1). Pooled samples were generated for *T. canis* and *T. cati*. Using a McMaster chamber, the egg concentrations in the pooled egg suspensions were determined and two-fold serial dilutions of 11 levels, starting at 1250 eggs/ml, were established and tested for both *T. canis* and *T. cati*. Siliconised (Sigmacote^®^, Sigma-Aldrich, Burlington, MA, USA) Falcon tubes were used. Three technical replicates of each dilution were prepared, and a negative control of 1 × PBS with 0.01% Tween20 was included in each case.

The sensitivity test for the parasitological methods was carried out with *T. cati* eggs, whereas for the DNA detection methods both *T. cati* and *T. canis* eggs were used separately. For SF, the prepared dilution steps were kept at 4 °C until examination. For mechanical lysis, zirconia beads were added to each egg suspension as described and the lysate was then extracted as described for KF24 and KF96. The egg suspensions of the dilution levels or lysates were stored at −20 °C until DNA extraction.

#### Diagnostic sensitivity using faecal samples

For the estimation of the diagnostic sensitivity, 300 faecal samples (Additional file 1: Table S1) were analysed with the same four methods.

### Calculation of the costs and processing time

Processing time and costs were estimated to examine a single sample and a set of 100 samples by each of the detection methods. The processing time estimate included both hands-on time and device processing. The cost estimate included consumables and reagents, but not equipment, laboratory, water, energy and personnel costs.

### Statistical testing

Statistical analyses and graphical presentations were performed using R version 4.1.3 [24]. Fisher’s exact test was used to test the null hypothesis of independence of rows and columns in a contingency table with fixed marginals as part of the R package “stats” [24]. The Wilcoxon rank sum test was used to test the difference between two dependent samples within the violin plot by “stat_compare_means” as part of the R package “ggpubr” [25]. Confidence intervals (95% CI) for each calculated proportion were calculated by R function “binom.test” within the R package “stats” [24]. Cohen’s kappa values were calculated by R function “CohenKappa” within the R package “DescTools” [26].

The limits of detection (LOD) of the different diagnostic methods were determined based on the results of the analytical sensitivity test. The LOD was defined as the lowest dilution step up to which *Toxocara* spp. could be consistently detected (at least two out of three replicates positive).

With the results of the field samples the diagnostic test accuracy was established with the methods described by Shim et al. [27] applying R package “meta” for univariate analyses and the R packages “mada”, “mvmeta”, “ellipse”, “mvtnorm” and “metafor” for bivariate analyses. For calculation of the positive and negative predictive values the R functions “ppv” or “npv” of the R package “yardstick” [28] were used, respectively.

For linear regression, the functions “stat_poly_line” and “stat_poly_eq” of the R package “ggplot2” [29] were used to display the regression lines and calculate the corresponding R^2^ values. The level of significance was calculated by the R function “lm” within the R package “stats” [24].

## Results

### Comparison of enzymatic and mechanical lysis for DNA extraction

**Fig. 2:**
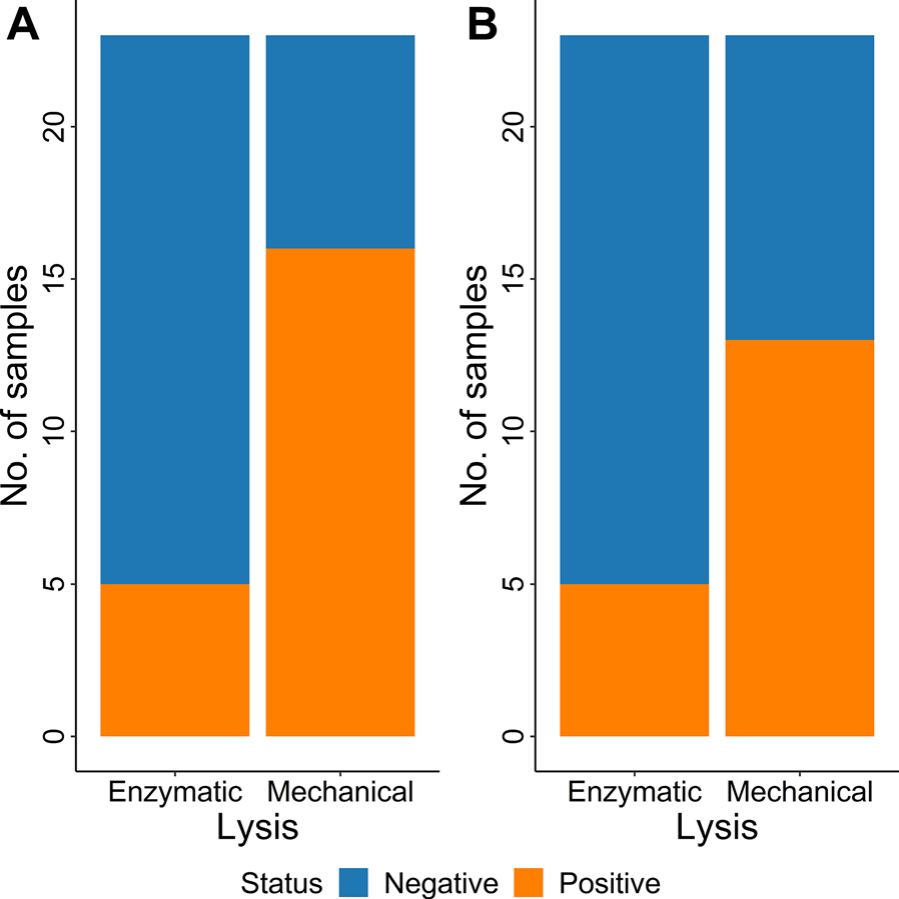
Comparison of enzymatic and mechanical lysis for four tested DNA extraction protocols. This comparison was performed by analysing extracted DNA by a multiplex real-time PCR for the simultaneous detection of *Toxocara canis* and *T. cati*. **A** DNA extracted with a King Fisher^®^ Flex System in 24-deep-well plates. The difference between positive results following enzymatic and mechanical lysis is significant (Fisher’s exact test, *P* = 0.003, OR = 0.1, 95% CI 0.03–0.54), **B** DNA extracted with a King Fisher^®^ Flex System in 96-well plates (Fisher’s exact test, *P* = 0.033, OR = 0.2, 95% CI 0.05–0.90)

The initial assessment, which involved 23 *Toxocara* spp.-positive faecal samples (Additional file 2: Table S2), provided evidence of a significantly higher sensitivity with mechanical lysis compared to enzymatic lysis. When the results for both species in the multiplex TC-qPCR were considered together, mechanical lysis was significantly more sensitive than enzymatic lysis for DNA extraction by both KF24 and KF96 (Fig. 2). For mechanical lysis and enzymatic lysis with KF24, 70.8% (95% CI 48.9%–87.4%) and 20.8% (95% CI 7.1%–42.2%) of the samples were positive, respectively. For KF96, 58.3% (95% CI 36.6%–77.9%) of samples were positive using mechanical lysis and 20.8% (95% CI 7.1%–42.2%) using enzymatic lysis (Additional file 2: Table S2, Fig. 2). For *T. canis*, mechanical lysis was significantly superior to enzymatic lysis in KF24 (Table 1). For KF24, focusing on *T. cati* results, and for KF96, focusing separately on results for *T. canis* or *T. cati*, the differences between mechanical and enzymatic lysis were not statistically significant (Table 1).

**Table 1:** Comparison of mechanical and enzymatic lysis for DNA extraction using 23 *Toxocara* spp.-positive faecal samples (2 cat and 21 dog samples); DNA was extracted with King Fisher® Flex System in 24- or 96-deep-well plates and amplified by a multiplex TC-qPCR for the simultaneous detection of *Toxocara canis* and *Toxocara cati*

DNA extraction	Species-specific detection in the multiplex TC-qPCR	No. of positive/total samples by mechanical lysis	No. of positive/total samples by enzymatic lysis	Fisher’s exact test *P*-value (OR, 95% CI)
DNA extraction with King Fisher^®^ Flex System in 24-deep-well plates	*T. canis*	10/23	3/23	0.047 (0.2, 0.03–0.98)
	*T. cati*	6/23	2/23	0.243 (0.3, 0.02–1.81)
DNA extraction with King Fisher^®^ Flex System in 96-well plates	*T. canis*	8/23	4/23	0.314 (0.4, 0.07–1.86)
	*T. cati*	5/23	1/23	0.187 (0.2, 0.003–1.71)

In dog samples, both *Toxocara canis* and *T. cati* were observed, while all cat samples tested *T. cati* positive only. Overall, samples extracted after mechanical lysis tested positive more often than samples extracted after enzymatic lysis

**Fig. 3:**
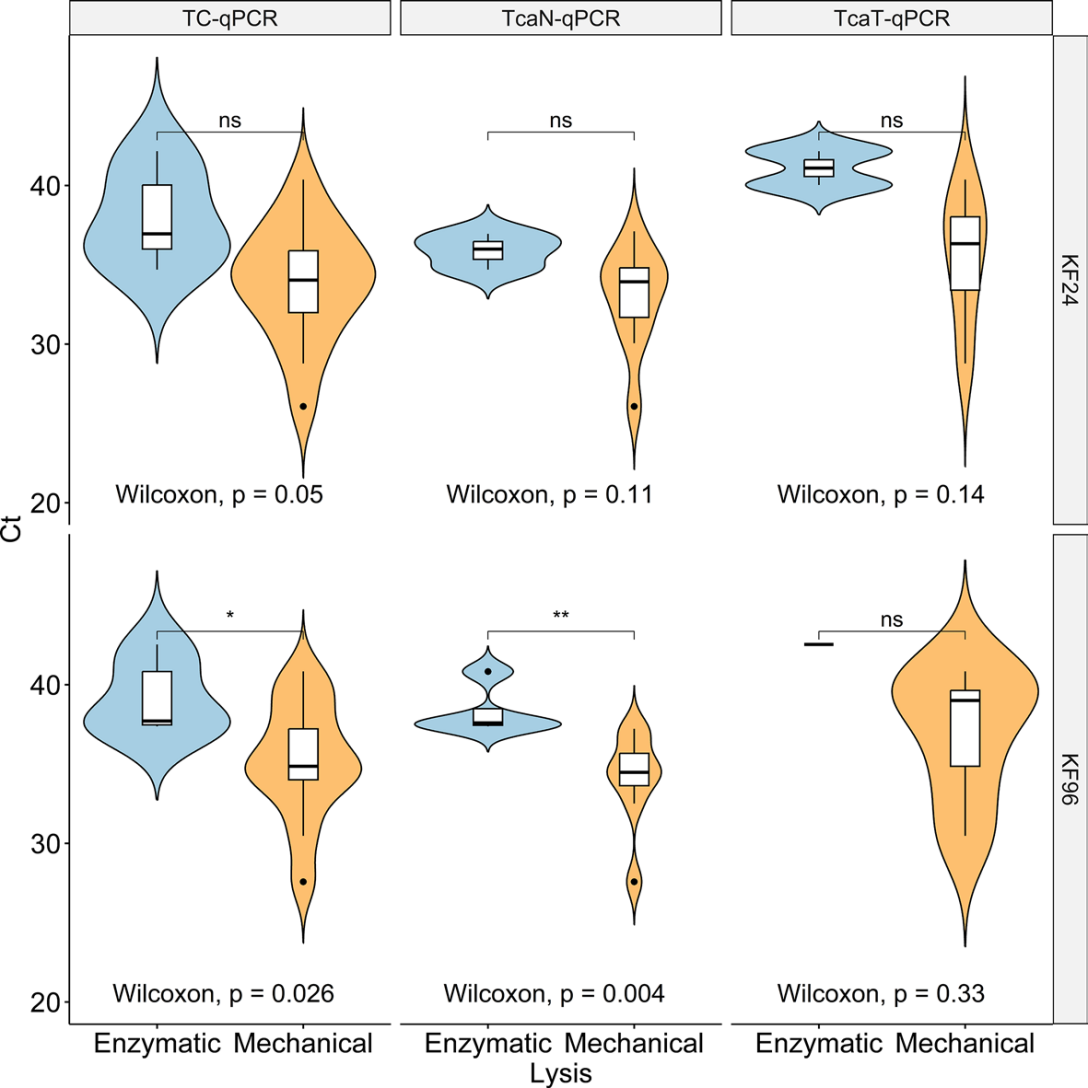
Comparison of Ct values obtained by four species-specific DNA detection protocols. This comparison is broken down according to lysis method. Extracted DNA was analysed by a multiplex real-time PCR for the simultaneous detection of *Toxocara canis* and *T. cati* (TC-qPCR). Comparisons between the performance of the lysis methods within each protocol are statistically analysed by Wilcoxon signed rank, with results displayed by a horizontal bar for the statistical significance level (ns: not significant, *P < 0.05, **P ≤ 0.01). KF24, DNA extraction with a King Fisher^®^ Flex System in 24-deep-well plates; KF96, DNA extraction with a King Fisher^®^ Flex System in 96-well plates; TcaN-qPCR, *T. canis*-specific results in the multiplex TC-qPCR; TcaT-qPCR, *T. cati*-specific results in the multiplex TC-qPCR

Furthermore, the quantity of *Toxocara* spp.-specific DNA obtained through enzymatic lysis tended to be lower compared to that obtained by mechanical lysis as reflected by differences in median Ct values (Fig. 3), but in most cases these differences were not significant.

Therefore, all further DNA detection methods explored in this study were performed with mechanical lysis.

### Analytical sensitivity of the used methods

**Fig. 4:**
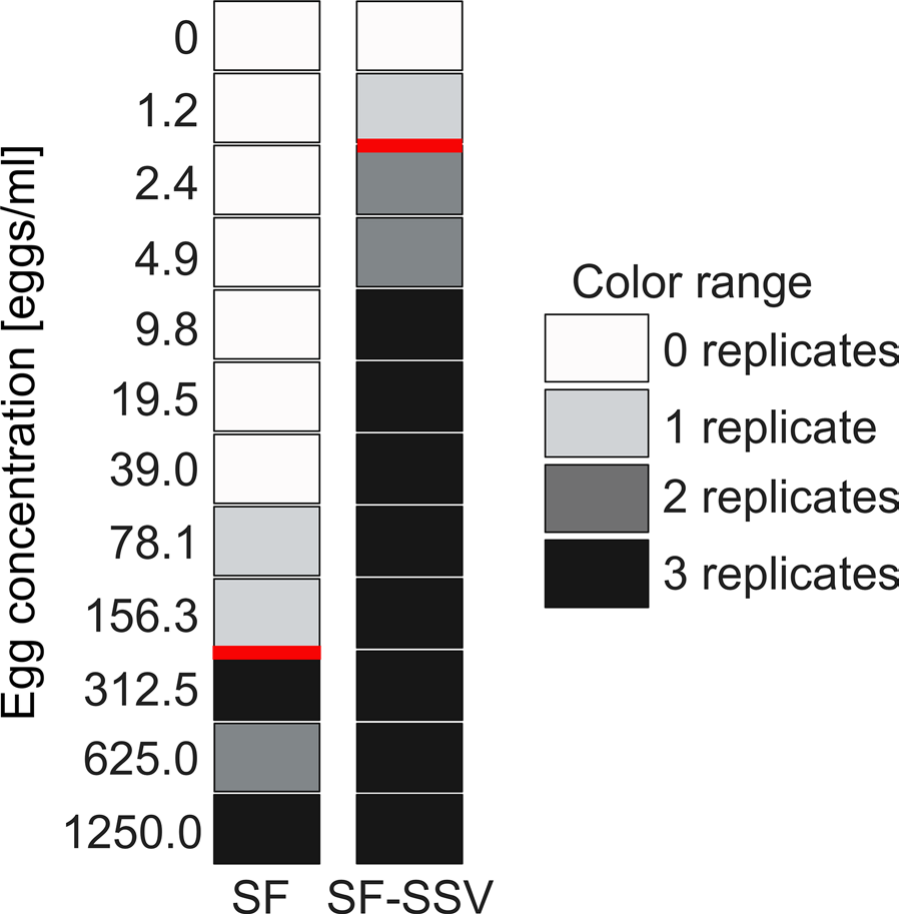
Analytical sensitivity of the SF and SF-SSV protocols. SF, sedimentation flotation technique; SF-SSV, sequential sieving method. The grey scale of the graph represents the number of positive replicates per egg concentration. Horizontal red lines indicate limits of detection. Created with BioRender.com

We assessed the LOD for the different methods employed or developed in this study. The SF revealed an LOD of 312.5 eggs/ml. In comparison, the SF-SSV showed a markedly higher analytical sensitivity, with an LOD of 2.4 eggs/ml (Fig. 4, Additional file 2: Table S3).

Application of the KF24 with *T. canis*-specific results in the multiplex TC-qPCR (KF24 + TcaN-qPCR) led to an LOD of 625.0 eggs/ml (equivalent to 17.4 eggs/extraction volume [1 ml]) and 78.1 eggs/ml for KF24 with *T. cati*-specific results in the multiplex TC-qPCR (KF24 + TcaT-qPCR; 2.17 eggs/extraction volume [1 ml]).

For KF96 with *T. canis*-specific results in the multiplex TC-qPCR (KF96 + TcaN-qPCR), the LOD was 156.3 eggs/ml (equivalent to 1.74 eggs/extraction volume [200 µl]) and for KF96 with *T. cati*-specific results in the multiplex TC-qPCR (KF96 + TcaT-qPCR), it was 39.0 eggs/ml (0.43 eggs/extraction volume [200 µl]).

**Fig. 5:**
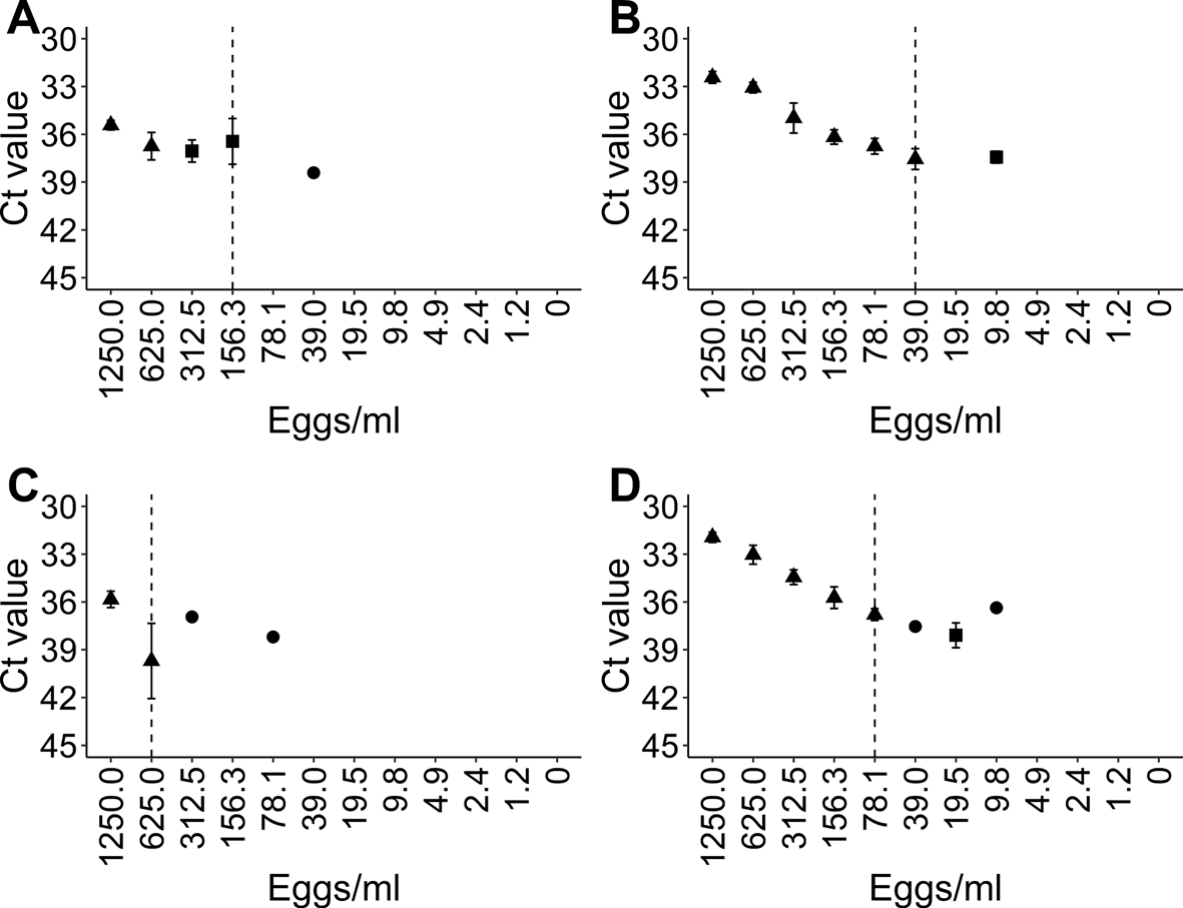
Mean Ct values calculated from three replicates for each concentration of serial egg dilution series. The multiplex real-time PCR results shown for the simultaneous detection of *Toxocara canis* and *T. cati* (TC-qPCR) are the outcome of the extraction of the dilution series using a King Fisher^®^ Flex System in 24- or 96-well plates (Additional file 2: Table S4). Dashed vertical lines indicate the respective limits of detection. Ct values (triangles, three positive replicates; squares, two positive replicates; dots, one positive replicate) are presented together with the respective standard deviation (whiskers) where applicable. **A** *Toxocara canis* results in the TC-qPCR for DNA extracted in 96-well plates, **B** *T. cati* results in the TC-qPCR for DNA extracted in 96-well plates, **C** *T. canis* results in the TC-qPCR for DNA extracted in 24-well plates, **D** *T. cati* results in the TC-qPCR for DNA extracted in 24-well plates

The LOD determination for the DNA detection methods was based on data presented in Fig. 5 (Additional file 2: Table S4, Additional file 2: Table S5). The SF-SSV exhibited the lowest LOD, i.e. the highest analytical sensitivity, when considering the sample volumes normalised to 1 ml (Table 2). However, the KF96 showed the highest analytical sensitivity when data from the actual sample volume analysed were used (Table 2).

**Table 2:** Analytical sensitivity of the analysed protocols

Parasitological or DNA extraction protocols	Species-specific detection in the multiplex TC-qPCR	Limit of detection by egg concentration	Limit of detection by number (sample volume used for testing)
Sedimentation flotation technique	NA	312.5 eggs/ml	312.5 eggs (1 ml)
Sequential sieving protocol	NA	2.4 eggs/ml	2.4 eggs (1 ml)
DNA extraction with King Fisher® Flex System in 24-deep-well plates	*Toxocara canis*	625.0 eggs/ml	17.4 eggs (1 ml)
	*Toxocara cati*	78.1 eggs/ml	2.17 eggs (1 ml)
DNA extraction with King Fisher® Flex System in 96-well plates	*T. canis*	156.3 eggs/ml	1.74 eggs (200 µl)
	*T. cati*	39.0 eggs/ml	0.43 eggs (200 µl)

The limit of detection for each protocol is expressed as eggs/volume. For the DNA detection methods, species-specific results in the multiplex TC-qPCR for the simultaneous detection of *Toxocara canis* and *Toxocara cati* are provided

*NA* not applicable

### Field samples

**Fig. 6:**
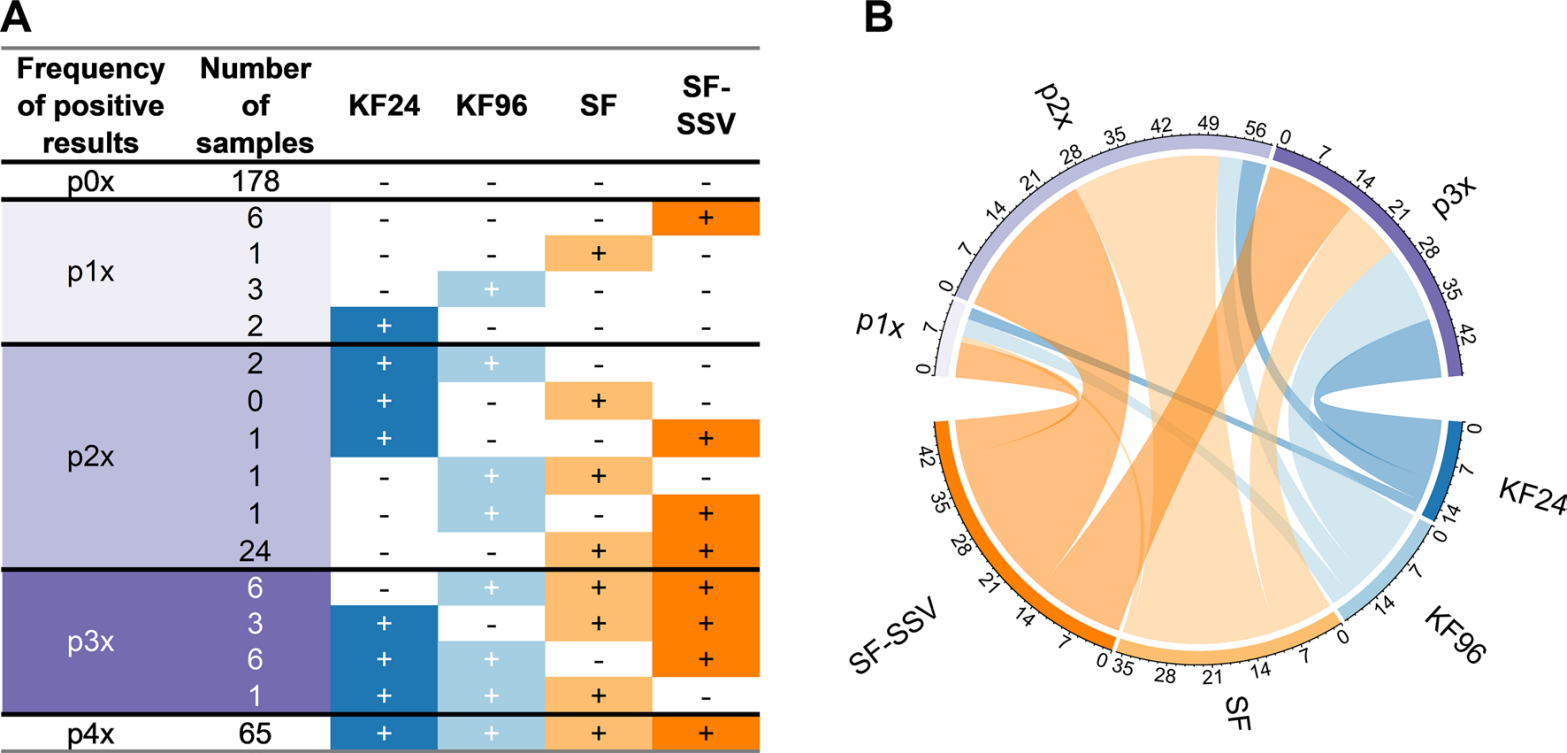
Results of the field faecal sample analysis. Number of field faecal samples (*n* = 300) tested positive (+) or negative (−) by four different *Toxocara* spp. detection protocols. These protocols include DNA extraction with a King Fisher^®^ Flex System in 24- or 96-well plates (KF24 or KF96, respectively), as well as the microscopy-based methods, sedimentation flotation (SF) and sequential sieving (SF-SSV). DNA samples were assessed by a multiplex real-time PCR for the simultaneous detection of *Toxocara canis* and *T. cati* (TC-qPCR). **A** The table shows combinations of positive and negative results by row using various protocols. **B** The distribution of the samples that were positive in one, two or three protocols is shown. The circos plot was created using the R package circlize [37]. *p1x* samples positive in one protocol, *p2x* samples positive in two protocols, *p3x* samples positive in three protocols

A total of 300 field samples (including 120 reference faecal samples and 180 samples from cats and dogs collected in the German Federal State of Mecklenburg-Western Pomerania) were examined by all four detection methods: SF, SF-SSV, KF24 and KF96 (including previous mechanical lysis and subsequent TC-qPCR, respectively). Results are shown in Table S1 (Additional file 1). Of these samples, 178 (59%) were negative across all methods, 65 (22%) were positive in all methods and the remaining 57 (19%) were positive in one, two or three methods. Figure 6 shows that 12 samples were positive by only one method of all applied protocols. For a further 29 samples, positive results were achieved in various combinations of two of the four methods (Fig. 6). Sixteen samples were positive in different combinations of three methods (Fig. 6). Only one possible combination was not met, that of being positive by KF24 and SF but simultaneously negative by KF96 and SF-SSV (Fig. 6).

The prevalence of *Toxocara* spp. in field samples from the SHIP NEXT project (*n* = 180) varied depending on the methods used (Table 3). When the *Toxocara* spp. prevalence in the tested population was stratified by host species, it varied for cats from 1.1% (95% CI 0.03%–6.2%) in KF24 to 6.9% (95% CI 2.6%–14.4%) in SF and SF-SSV. For dogs, the prevalence ranged from 1.1% (95% CI 0.03%–5.8%) in KF24 to 6.5% (95% CI 2.4%–13.5%) in SF-SSV.

**Table 3:** Prevalence of *Toxocara* spp. using 180 faecal samples collected from cats and dogs in the German Federal State of Mecklenburg-Western Pomerania

Host species	Parasite species	Proportion of positive findings according to method [95% CI]			
		KF24 + TC-qPCR	KF96 + TC-qPCR	SF	SF-SSV
Cats	*T. canis*, *T. cati*	1.1% [0.03%–6.2%]	3.4% [0.7%–9.7%]	6.9% [2.6%–14.4%]	6.9% [2.6%–14.4%]
	*T. canis*	–	–	NA	NA
	*T. cati*	1.1% [0.03%–6.2%]	3.4% [0.7%–9.7%]	NA	NA
Dogs	*T. canis*, *T. cati*	1.1% [0.03%–5.8%]	4.3% [1.2%–10.6%]	3.2% [0.7%–9.1%]	6.5% [2.4%–13.5%]
	*T. canis*	1.1% [0.03%–5.8%]	1.1% [0.03%–5.8%]	NA	NA
	*T. cati*	1.1% [0.03%–5.8%]	4.3% [1.2%–10.6%]	NA	NA

*KF24* DNA extraction with King Fisher® Flex System in 24-deep-well plates (including previous mechanical lysis and subsequent TaqMan® real-time PCR for *Toxocara canis* and *T. cati*), *KF96* DNA extraction with King Fisher® Flex System in 96-well plates (including mechanical lysis and TaqMan® real-time PCR for *T. canis* and *T. cati*), *SF* sedimentation flotation technique, *SF-SSV* sequential sieving method, *NA* not applicable

When analysing the TC-qPCR results, where it was possible to distinguish the *Toxocara* species, a prevalence of 1.1% (95% CI 0.03%–5.8%) for *T. canis* and 4.3% (95% CI 1.2%–10.6%) for *T. cati* was found in dogs. For cats, infection with *T. cati* was detected in 3.4% (95% CI 0.7%–9.7%) of the animals. There were no *T. canis*-positive findings in cats (Table 3).

### Pairwise agreements of the methods

To assess pairwise agreement between the methods applied to the field samples, Cohen’s kappa values were calculated and classified [30]. There was almost perfect agreement between the results of SF and SF-SSV examinations of the field samples (Cohen’s kappa value: κ = 0.88 [95% CI 0.82–0.93]). A similar outcome was observed for KF24 and KF96 (Cohen’s kappa value: κ = 0.86 [95% CI 0.79–0.92]). Yet, when the conventional parasitological methods (SF and SF-SSV) were compared to the DNA detection methods (KF24 + TC-qPCR and KF96 + TC-qPCR), there was only substantial agreement (Cohen’s kappa value: κ = 0.72 [95% CI 0.64–0.80]).

### Diagnostic test accuracy

Diagnostic test accuracy was analysed based on the number of true positives, true negatives, false positives and false negatives detected by each method (Table 4). For the purpose of this analysis, the following definitions were used to account for the lack of a gold standard: samples positive by any of the four methods were defined as true positives and samples negative by all four methods were defined as true negatives. Hence, it was assumed that no sample was false positive in any of the applied methods.

**Table 4:** True-positive as well as true- and false-negative results in four diagnostic methods performed on the field samples (*n* = 300)

Method	True positive	False negative	True negative
KF24	80	42	178
KF96	85	37	178
SF	101	21	178
SF-SSV	112	10	178

The samples were assumed as true positive if tested positive by at least one method. False-positive samples do not exist in this analysis according to the assumption mentioned above

*KF24* DNA extraction with King Fisher® Flex System in 24-deep-well plates (including previous mechanical lysis and subsequent TaqMan® real-time PCR for *Toxocara canis* and *T. cati*), *KF96* DNA extraction with King Fisher® Flex System in 96-well plates (including previous mechanical lysis and subsequent TaqMan® real-time PCR for *T. canis* and *T. cati*), *SF* sedimentation flotation technique, *SF-SSV* sequential sieving method

In the course of the diagnostic test accuracy analysis, diagnostic sensitivity, diagnostic specificity, positive and negative predictive values as well as diagnostic odds ratios (DORs) were estimated. The resulting estimates differed between the compared methods (Table 5). The differences between SF-SSV with the highest diagnostic sensitivity (91.8% [95% CI 85.4%–96.0%]) and the DNA detection methods with lower diagnostic sensitivities (KF96 69.7% [95% CI 60.7%–77.7%] and KF24 65.6% [95% CI 56.4%–73.9%]) were statistically significant. The difference between SF (82.8% [95% CI 74.9%–89.0%]) and KF24 was also significant.

**Table 5:** Results of the accuracy analysis for the four diagnostic protocols used for the examination of field samples

Method	Sensitivity [95% CI]	Specificity [95% CI]	Positive predictive value	Negative predictive value
KF24	65.6% [56.4%–73.9%]	100.0% [97.0%–100.0%]	100.0%	80.9%
KF96	69.7% [60.7%–77.7%]	100.0% [97.0%–100.0%]	100.0%	82.8%
SF	82.8% [74.9%–89.0%]	100.0% [97.0%–100.0%]	100.0%	89.4%
SF-SSV	91.8% [85.4%–96.0%]	100.0% [97.0%–100.0%]	100.0%	94.7%

*KF24* DNA extraction with King Fisher® Flex System in 24-deep-well plates (including previous mechanical lysis and subsequent TaqMan® real-time PCR for *Toxocara canis* and *T. cati*), *KF96* DNA extraction with King Fisher® Flex System in 96-well plates (including previous mechanical lysis and subsequent TaqMan® real-time PCR for *T. canis* and *T. cati*), *SF* sedimentation flotation technique, *SF-SSV* sequential sieving method

Due to the chosen assumptions, the estimates for specificity and the positive predictive values of all methods were necessarily 100% (Table 5). However, the negative predictive values for these methods in the field population could be calculated and ranged from 80.9% (KF24) to 94.7% (SF-SSV) (Table 5). We considered calculating a DOR, but due to the assumptions made to account for the lack of a gold standard, we reasoned that such a calculation would be inappropriate.

### Comparison between Ct values and egg numbers in field samples

To determine whether the egg burden can be predicted by Ct values of the respective DNA detection method, the correlation between the number of eggs counted by parasitological methods and the respective Ct values was analysed.

**Fig. 7:**
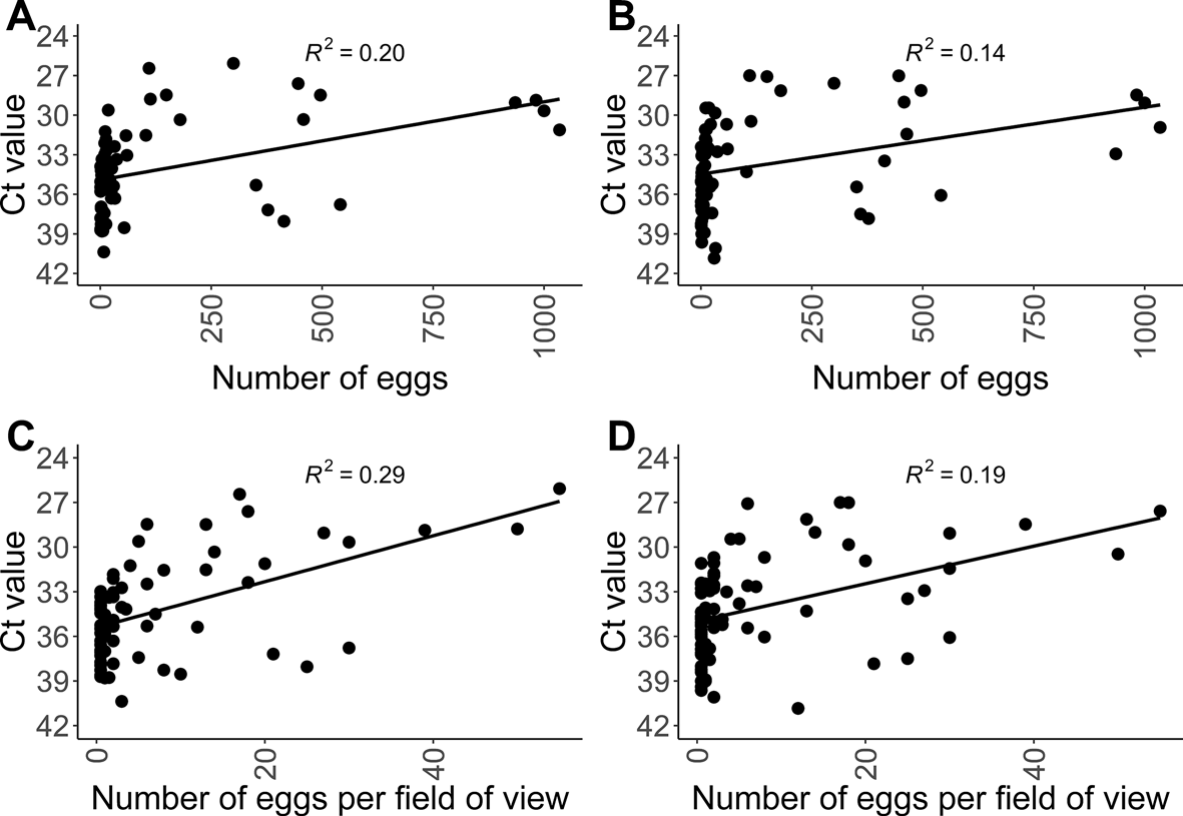
Comparison of results by molecular biological and parasitological protocols. Results by a multiplex real-time PCR (Ct) for the simultaneous detection of *Toxocara canis* and *T. cati* regarding egg counts or number of eggs per field of microscopic view as determined by sedimentation flotation (SF) and sequential sieving (SF-SSV), respectively. The different terms are related to the different ways of egg counting. For SF, the entire slide was counted, whereas for SF-SSV ten fields of view were counted, with the mean value of this count provided (number of eggs per field of view). The lines represent linear regressions, which were calculated by the function “stat_poly_line”. Another function, “stat_poly_eq”, of the R package “ggplot2” [29] was used to calculate the corresponding R^2^ values. **A** Comparison of KF24 against SF, **B** comparison of KF96 against SF, **C** comparison of KF24 against SF-SSV, **D** comparison of KF96 against SF-SSV. *KF24* DNA extraction with King Fisher® Flex System in 24-deep-well plates, *KF96* DNA extraction with King Fisher^®^ Flex System in 96-well plates

The statistically significant correlation with the highest coefficient of determination (R^2^ = 0.29, *P* < 0.0001) was found between the Ct values determined by KF24 and number of eggs determined by SF-SSV. The statistically significant correlation with the lowest coefficient of determination (R^2^ = 0.14, *P* = 0.001) was between KF96 and SF (Fig. 7).

### Cost and processing time of the used methods

Decision-making regarding a diagnostic method for a particular project or purpose is influenced not only by the performance of the method but also by the costs and processing times associated with the different protocols. To this end, an attempt was made to calculate the costs and processing times associated with each of the protocols under examination. Not surprisingly, the costs and time required for processing were non-linearly dependent on the number of samples processed (Table 6). For individual samples, the costs for the DNA detection methods (KF24 + TC-qPCR: 30.83 €, KF96 + TC-qPCR: 27.74 €) were markedly higher than for the parasitological methods (SF: 0.41 €, SF-SSV: 6.00 €). When 100 samples were considered, the cost per sample for both parasitological methods, SF and SF-SSV, remained largely unchanged (0.41 € and 6.00 €, respectively), whereas the cost per sample for the PCR detection methods decreased to become comparable with that of SF-SSV (KF96 + TC-qPCR: 5.72 € and KF24 + TC-qPCR: 8.11 €) (Table 6).

**Table 6:** Cost and processing time of conventional parasitological and DNA detection methods used to detect *Toxocara* spp. eggs in faecal samples

	SF	SF-SSV	KF24 + TC-qPCR	KF96 + TC-qPCR
Costs^a^/sample for one sample (€)	0.41	6.00	30.83	27.74
Costs^a^/sample for 100 samples (€)	0.41	6.00	8.11	5.72
Total time expenditure for one sample^b^ (h)	1.2	1.5	3.7	3.7
Total time expenditure for 100 samples^b^ (h)	55.0	101.6	29.9	19.1

^a^Only costs of consumables (e.g. centrifuge tubes, pipette tips) and reagents (e.g. DNA extraction kit, real-time PCR kit) were included. Equipment costs (e.g. cycler, centrifuge), personnel costs and costs for electricity and water were not included. The prices used for the calculation refer to the purchase prices of a major laboratory supplier in the year 2023

^b^Approximate processing time, depending on the operator

A similar non-linear relationship was observed for the time required to perform the methods. For single samples, the parasitological methods (SF: 1.2 h, SF-SSV: 1.5 h) required less time than the DNA detection methods (3.7 h). However, when 100 samples were considered, the time saving was especially noticeable, with the DNA detection methods requiring only 19.1 h (KF96 + TC-qPCR) and 29.9 h (KF24 + TC-qPCR) in contrast to the conventional parasitological methods that required 55.0 h (SF) and 101.6 h (SF-SSV) in total.

## Discussion

This study sought to compare the analytical and diagnostic sensitivity of microscopy-based parasitological (SF, SF-SSV) and high-throughput DNA detection protocols. The latter aimed to facilitate large epidemiological studies and to detect parasites at the species level (KF96 + TC-qPCR; KF24 + TC-qPCR), i.e. by applying a multiplex qPCR for the simultaneous detection of *T. canis* and *T. cati*. While SF-SSV showed the highest analytic and diagnostic sensitivities, KF96 + TC-qPCR showed lower but acceptable sensitivities.

Cost and time necessary to analyse samples were additional factors compared in this study. SF, followed by SF-SSV, proved to be the most cost-effective technique, but with higher sample numbers, these conventional parasitological methods became highly time-consuming. In contrast, for the DNA detection methods, the costs per sample considerably decreased when 100 samples were analysed instead of a single sample, and the time required to process 100 samples was lower compared to the microscopy-based parasitological methods. For this reason, the KF96 + TC-qPCR method may become an alternative for epidemiological studies in which large numbers of samples are examined and where the species differentiation of *T. canis* and *T. cati* is of importance.

Regarding the conventional parasitological methods, the SF-SSV approach appeared to be a promising alternative to SF given its higher analytic and diagnostic sensitivity. This finding was not surprising considering that nearly the entire flotation solution is used for SF-SSV, whereas only material floating on the surface of the solution is used for SF examination. In addition, SF-SSV also removes particles that obstruct vision, both large and small, which can ease microscopical examination and subsequently improve sensitivity.

SF-SSV was found to have a higher diagnostic sensitivity compared to the DNA detection methods. Copro-inhibitors present in faecal samples may have had a negative effect on the multiplex TC-qPCR. However, amplification of the internal control integrated into the qPCR argued against inhibition as a potential reason; in addition, a specific additive was given to the extracted DNA (i.e. the S.T.A.R. buffer) to minimise possible inhibition. Another reason for the lower diagnostic sensitivity of the DNA detection methods could be the out-competition of parasite DNA during extraction, as any DNA (i.e. non-specific faecal DNA, including host DNA) can bind to the beads used for extraction, leaving less or even no binding capacity for the *Toxocara* spp.-specific DNA [31]. Since faecal samples represent a complex matrix, further effects seem possible, such as a change in pH or in the concentration of interfering compounds, which may have overloaded the buffering capacity of the S.T.A.R. buffer.

For the DNA detection methods, two different lysate volumes, 200 µl (KF96) and 1000 µl (KF24), were used in the DNA extraction step. Surprisingly, the logical assumption that using a larger lysate volume in KF24 should lead to a higher analytical sensitivity than in KF96 was not supported by the results. The KF96 approach was always superior regarding both analytical and diagnostic sensitivity. A potential reason for this unexpected result could be that a larger lysate volume translates into a larger amount of DNA, regardless of whether it is parasite DNA or other DNA present in faecal matter. Therefore, the possibility of an out-competition of the *Toxocara* spp.-specific DNA by faecal DNA represents a possible explanation for this observation, given the limited binding capacity of the beads used for extraction.

Although the investigation of pairwise agreement of the results for parasite detection in the field samples showed substantial agreement between the microscopy-based parasitological and the DNA detection methods, the examination of possible correlations between the Ct values of the TC-qPCR and the counted egg numbers in the SF and SF-SSV showed coefficients of determination no greater than 29%. These low coefficients of determination could be partially attributed to variations in the copy number of the diagnostic target (the internal transcribed spacer 2 sequence of the ribosomal DNA) per egg, which likely depends on the developmental stage of the egg at the time of DNA extraction [32]. This illustrates that DNA detection only partially reflects the results of the microscopy-based parasitological methods and shows that both molecular and microscopy-based methods may have considerable limitations. This is further supported by the observation that several instances of divergent results occurred when field samples were tested with the different diagnostic methods. There are many possible explanations for why several samples were positive in some methods and not in others. One possibility is that eggs were not homogeneously distributed in various portions of a sample [33], even after homogenisation. Another possibility is that the *Toxocara* spp. eggs were completely or partially destroyed or damaged, e.g. by the freezing process applied for biosafety purposes in this study. This may have made them difficult or impossible to detect by microscopy but still able to be detected by methods targeting the genome of the parasites.

A gold-standard for estimating the diagnostic test accuracy was lacking. Therefore, a sample was considered positive when visible eggs were present and/or pathogen DNA could be detected by the TC-qPCR. Consequently, it was assumed that there were no false-positive samples in the validation set. We considered this a reasonable assumption for the following reasons. The specificity of the qPCR was reported as 100% for *T. canis* and 95.8% for *T. cati* [16]. While there is a lack of reported information about the specificity of microscopic examination, *Toxocara* spp. eggs are generally easy to recognise, so false-positive findings are uncommon. The samples at greatest risk of misclassification (those that tested positive by only one of the four detection methods) represent only 4% of the total sample collection. These points suggest that whilst misclassification resulting from our assumption cannot be ruled out and could bias the results, the impact on the overall findings would be minimal. With this justified approach, various mean diagnostic sensitivities were estimated to range from 65.6% to 69.7% for DNA detection and from 82.8% to 91.8% for conventional parasitological methods. The estimate reached for SF (82.8%) was comparable to the value of 87% reported in the literature [12]. For the other methods, no reference values were available.

Analysis of the cost and time required for each of the diagnostic methods showed that the microscopy-based protocols are highly cost- and time-effective if only a few samples are tested, but for larger sample numbers, the DNA detection protocols, especially KF96 + TC-qPCR, out-performed the microscopy-based protocols. The DNA detection methods have additional noteworthy advantages over the microscopy-based protocols: No specifically trained staff are needed (thus also reducing the risk of operator bias), the analytic process can be automated, and species-specific diagnosis can be performed, i.e. differentiation between *T. canis* and *T. cati*. These techniques may find use in larger epidemiological studies in which knowledge on the possibly lower diagnostic sensitivity can be compensated for by including the data on the estimated diagnostic sensitivity in the data analysis [34].

The importance of a species-specific diagnosis is illustrated by the fact that without a differentiation between the two *Toxocara* species by the multiplex TC-qPCR, it would have been missed that dogs shed not only *T. canis* but also *T. cati*. A possible explanation for this is the coprophagic behaviour of dogs [35]. As *T. cati* eggs may remain infectious despite intestinal passage in dogs, this finding is of considerable zoonotic concern [35]. At the same time, *T. canis* findings in dog faeces also require careful interpretation, as these eggs could be caused by consumption of faeces from foxes and other dogs [35]. Enzyme-linked immunosorbent assays (e.g. IDEXX Fecal Dx® antigen tests) can clarify the infection status of dogs as they detect coproantigens of adult worms in the faeces [36].

In our study, the overall estimated prevalence of *Toxocara* spp. in Mecklenburg-Western Pomerania was 6.9% (95% CI 2.6%–14.4%) in cats and 6.5% (95% CI 2.4%–13.5%) in dogs based on the results obtained with the most sensitive tests. This was higher than, or similar to, those reported in other studies within Germany of 0.0% to 3.9% [8], 3.5% to 4.8% [9] or 7.7% [10] in cats and 0.9% to 6.1% [8], 3.8% to 4.6% [9] or 5.9% [11] in dogs.

## Conclusions

The most sensitive methods for the detection of *Toxocara* spp. in faecal samples of cats and dogs were found to be the classical microscopy-based diagnostic techniques, specifically SF-SSV. However, multiplex qPCR-based DNA detection may represent a good alternative method, particularly in situations where large numbers of samples need to be processed, where personnel specifically trained in the microscopical detection of *Toxocara* spp. eggs is lacking and/or where species differentiation is necessary.

## Supplementary Information

Additional file 1: Table S1. Raw data of the 300 faecal samples including host species, results of the diagnostic protocols tested and labelling for which parts of the study the samples were included.

Additional file 2: Table S2. Results of comparison between enzymatic and mechanical cell lysis with KF24 and KF96 and subsequent real-time PCR for both *Toxocara canis* and *T. cati*. Table S3. Results of analytical sensitivity test from the parasitological methods. Table S4. Results of analytical sensitivity test from DNA detection with King Fisher^®^ Flex System in 96-well plates and subsequent multiplex real-time PCR for *Toxocara canis* and *T. cati*. Table S5. Results of analytical sensitivity test from DNA extraction with King Fisher^®^ Flex System in 24-deep-well-plates and subsequent multiplex real-time PCR for *Toxocara canis* and *T. cati*.

## Data Availability

No datasets were generated or analysed during the current study.

## Abbreviations

DOR: Diagnostic odds ratios
KF96 + TcaT-qPCR: DNA extraction with King Fisher^®^ Flex System in 96-well plates in combination with *Toxocara cati*-specific results in the multiplex TaqMan^®^ real-time PCR
LOD: Limit of detection
qPCR: TaqMan^®^-based real-time quantitative PCR
RT: Room temperature
SHIP: Study of Health in Pomerania
SF: Sedimentation-flotation technique
SF-SSV: Sedimentation-flotation technique with subsequent sequential sieving protocol
SSV: Sequential sieving protocol
TC-qPCR: TaqMan® real-time PCR for *Toxocara canis* and *T. cati*
TcaN-qPCR: *Toxocara canis*-specific results in the multiplex TaqMan^®^ real-time PCR
TcaT-qPCR: *Toxocara cati*-specific results in the multiplex TaqMan^®^ real-time PCR
95% CI: Confidence interval (95%)
