# Supplementary material for: Comparison of different diagnostic protocols for the detection of Toxocara spp. in faecal samples of cats and dogs
Source: Parasit Vectors. 2024 Oct 24;17:436. doi: 10.1186/s13071-024-06524-x (PMC11515329; doi:10.1186/s13071-024-06524-x)
Supplement: Supplementary file 2 — Additional file 2: Table S2. Results of comparison between enzymatic and mechanical cell lysis with KF24 and KF96 and subsequent real-time PCR for both Toxocara canis and T. cati. Table S3. Results of analytical sensitivity test from the parasitological methods. Table S4. Results of analytical sensitivity test from DNA detection with King Fisher® Flex System in 96-well plates and subsequent multiplex real-time PCR for Toxocara canis and T. cati. Table S5. Results of analytical sensitivity test from DNA extraction with King Fisher® Flex System in 24-deep-well-plates and subsequent multiplex real-time PCR for Toxocara canis and T. cati. [file 13071_2024_6524_MOESM2_ESM.docx]

Table S2 Results of comparison between enzymatic and mechanical cell lysis with KF24 and KF96 and subsequent real-time PCR for both Toxocara canis and Toxocara cati.

|  |  | Ct-values | | | | | | | | SF |
| --- | --- | --- | --- | --- | --- | --- | --- | --- | --- | --- |
|  |  | KF24 mechanical lysis | | KF96 mechanical lysis | | KF24 enzymatic lysis | | KF96 enzymatic lysis | |  |
| Nr. | Host species | *T. canis* | *T. cati* | *T. canis* | *T. cati* | *T. canis* | *T. cati* | *T. canis* | *T. cati* |  |
| 1 | dog | 35.48 | - | 36.86 | - | - | - | - | - | - |
| 2 | dog | - | 37.28 | - | 39.01 | - | - | - | - | - |
| 3 | dog | 37.11 | - | - | - | - | - | 40.83 | - | - |
| 4 | cat | - | 35.38 | - | 40.84 | - | - | - | - | ++ |
| 5 |  | - | 40.37 | - | - | - | - | - | - | + |
| 6 | dog | - | 32.74 | - | 34.86 | - | 42.16 | - | - | ++ |
| 7 | dog | 32.14 | - | 34.01 | - | 35.99 | - | 37.71 | - | - |
| 8 | cat | - | 28.78 | - | 30.47 | - | 40.03 | - | 42.54 | +++ |
| 9 | dog | - | - | - | - | - | - | - | - | + |
| 10 | dog | - | - | - | - | - | - | - | - | + |
| 11 | dog | 31.53 | - | 34.30 | - | - | - | - | - | +++ |
| 12 | dog | 35.04 | - | - | - | - | - | 37.37 | - | + |
| 13 | dog | 33.95 | - | 35.28 | - | - | - | - | - | + |
| 14 | dog | - | - | - | - | - | - | - | - | - |
| 15 | dog | - | - | - | - | - | - | - | - | - |
| 16 | dog | - | - | - | - | - | - | - | - | - |
| 17 | dog | 30.05 | - | 32.51 | - | - | - | - | - | - |
| 18 | dog | 33.91 | - | 34.66 | - | - | - | 37.47 | - | + |
| 19 | dog | 34.12 | - | 37.22 | - | 34.69 | - | - | - | + |
| 20 | dog | 26.07 | - | 27.58 | - | 36.95 | - | - | - | +++ |
| 21 | dog | - | 38.27 | - | 39.64 | - | - | - | - | + |
| 22 | dog | - | - | - | - | - | - | - | - | ++ |
| 23 | dog | - | - | - | - | - | - | - | - | + |

KF24, DNA extraction with King Fisher® Flex System in 24-deep-well-plates (including subsequent TaqMan® real-time PCR for *Toxocara canis* and *Toxocara cati*); KF96, DNA extraction with King Fisher® Flex System in 96-well-plates (including subsequent TaqMan® real-time PCR for *T. canis* and *T. cati*); SF, Sedimentation flotation technique; +, < 10 eggs; ++, 10 - 100 eggs; +++, > 100 eggs; -, 0 eggs or no detected DNA.

Table S3 Results of analytical sensitivity test from the parasitological methods.

| Calculated Concentration [eggs/ml] | 1250 | 625 | 312,5 | 156,3 | 78,1 | 39 | 19,5 | 9,8 | 4,9 | 2,4 | 1,2 | 0 |
| --- | --- | --- | --- | --- | --- | --- | --- | --- | --- | --- | --- | --- |
| Sedimentation-flotation replicate 1 | ++ | + | + | - | + | - | - | - | - | - | - | - |
| Sedimentation-flotation replicate 2 | + | - | + | + | - | - | - | - | - | - | - | - |
| Sedimentation-flotation replicate 3 | + | ++ | + | - | - | - | - | - | - | - | - | - |
| Sieving method replicate 1 | + | + | + | + | + | + | + | + | + | - | - | - |
| Sieving method replicate 2 | + | + | + | + | + | + | + | + | + | + | - | - |
| Sieving method replicate 3 | + | + | + | + | + | + | + | + | - | + | + | - |

+ = positive for *Toxocara* spp. eggs (Sieving method), + = < 10 eggs (Sedimentation flotation), ++ = 10 - 100 eggs (Sedimentation flotation), - = 0 eggs

Table S4 Results of analytical sensitivity test from DNA detection with King Fisher® Flex System in 96-well-plates and subsequent multiplex real-time PCR for Toxocara canis and Toxocara cati.

| Calculated Concentration per extraction volume [eggs/200 µl] | 13, 89 | 6,94 | 3,47 | 1,74 | 0,87 | 0,43 | 0,22 | 0,11 | 0,05 | 0,03 | 0,01 | 0 |
| --- | --- | --- | --- | --- | --- | --- | --- | --- | --- | --- | --- | --- |
| DNA extraction KF96 replicate 1 C_t_-Wert T. canis | 35.08 | 37.74 | NA | 37.46 | NA | NA | NA | NA | NA | NA | NA | NA |
| DNA extraction KF96 replicate 2 C_t_-Wert T. canis | 35.57 | 36.19 | 37.54 | NA | NA | NA | NA | NA | NA | NA | NA | NA |
| DNA extraction KF96 replicate 3 C_t_-Wert T. canis | 35.63 | 36.30 | 36.56 | 35.43 | NA | 38.42 | NA | NA | NA | NA | NA | NA |
| DNA extraction KF96 replicate 1 C_t_-Wert T. cati | 32.85 | 32.70 | 34.07 | 35.70 | 36.39 | 37.10 | NA | 37.17 | NA | NA | NA | NA |
| DNA extraction KF96 replicate 2 C_t_-Wert T. cati | 32.25 | 33.35 | 35.95 | 36.21 | 36.54 | 37.26 | NA | 37.70 | NA | NA | NA | NA |
| DNA extraction KF96 replicate 3 C_t_-Wert T. cati | 32.17 | 33.15 | 34.91 | 36.60 | 37.31 | 38.30 | NA | NA | NA | NA | NA | NA |

NA, not applicable.

Table S5 Results of analytical sensitivity test from DNA extraction with King Fisher® Flex System in 24-deep-well-plates and subsequent multiplex real-time PCR for Toxocara canis and Toxocara cati.

| Calculated Concentration per extraction volume [eggs/1 ml] | 34,72 | 17,36 | 8,68 | 4,34 | 2,17 | 1,08 | 0,54 | 0,27 | 0,14 | 0,07 | 0,03 | 0 |
| --- | --- | --- | --- | --- | --- | --- | --- | --- | --- | --- | --- | --- |
| DNA extraction KF24 replicate 1 C_t_-Wert T. canis | 36.24 | 42.43 | NA | NA | NA | NA | NA | NA | NA | NA | NA | NA |
| DNA extraction KF24 replicate 2 C_t_-Wert T. canis | 36.02 | 38.24 | 36.94 | NA | NA | NA | NA | NA | NA | NA | NA | NA |
| DNA extraction KF24 replicate 3 C_t_-Wert T. canis | 35.26 | 38.44 | NA | NA | 38.20 | NA | NA | NA | NA | NA | NA | NA |
| DNA extraction KF24 replicate 1 C_t_-Wert T. cati | 32.04 | 32.40 | 34.02 | 35.15 | 37.01 | NA | NA | NA | NA | NA | NA | NA |
| DNA extraction KF24 replicate 2 C_t_-Wert T. cati | 32.19 | 33.12 | 34.94 | 36.48 | 37.01 | 37.54 | 37.54 | NA | NA | NA | NA | NA |
| DNA extraction KF24 replicate 3 C_t_-Wert T. cati | 31.58 | 33.58 | 34.37 | 35.56 | 36.37 | NA | 38.64 | 36.37 | NA | NA | NA | NA |

NA, not applicable.
